# Do Global Adolescents With Food Insecurity Feel Lonely?

**DOI:** 10.3389/fpubh.2022.820444

**Published:** 2022-02-10

**Authors:** Haowen Wu, Zhijun Gu, Linmiao Zeng, Tianyou Guo

**Affiliations:** ^1^School of Government, Institute of Urban Governance, Shenzhen University, Shenzhen, China; ^2^School of Public Administration and Emergency Management, Jinan University, Guangzhou, China; ^3^Exercise Psychophysiology Laboratory, Institute of KEEP Collaborative Innovation, School of Psychology, Shenzhen University, Shenzhen, China

**Keywords:** adolescent, food insecurity, socioeconomic status, loneliness, GSHS

## Abstract

As a proxy measure of socioeconomic status, food insecurity is understudied in mental health-related research. This study aimed to explore the association between food insecurity and loneliness in adolescents. Using cross-sectional data from the Global Student Health Survey (GSHS), 164,993 adolescent participants were included in this study. Food insecurity, loneliness, and other covariates were assessed by self-reported questionnaire. Multivariable logistics regression considering complex survey was used to explore the association between food insecurity and loneliness. The prevalence of loneliness was 10.8% in adolescents. With higher levels of food insecurity, the prevalence of loneliness in general increased, but “most of the time” was the most frequently reported item in terms of food insecurity. Adolescents who reported severe food insecurity had significantly greater odds for loneliness: (1) being most of the time [odd ratio (OR) = 2.54, 95% CI = 2.13–3.02]; (2) always hungry (OR = 1.97, 95% CI = 1.55–2.51). Of all the 53 countries, adolescents from 39 countries reported significantly higher prevalence of loneliness when exposed to food insecurity. The pooled OR was 1.74 (1.60–1.89) with a negligible heterogeneity (higher I-squared was 34.2%). Adolescents with food insecurity were more likely to be exposed to be lonely. Eliminating socioeconomic disparities in adolescents might be a good approach to promote mental health in adolescents. Future studies are encouraged to utilize longitudinal studies to confirm or negate our study findings.

## Introduction

Loneliness is the unpleasant emotional state in response to social isolation and poor social connection (1, 2). Loneliness has become a public health concern, as the prevalence has reached approximately 11% in the general population in 2017 (3), resulting in both physical and mental illnesses (4). For example, several previous studies indicate loneliness-related adverse effects on elevating blood pressure, weakening the immune system as well as such mental problem was linked to the greater possibility of developing diabetes, dementia, and depressive and anxiety disorders. Of note, theses mental problems have been commonly reported among older individuals, immigrants, LGBT, and violence victims (5), for example, poor social relationship or social isolation (loneliness) of adults over 50 and older faced 50, 29 and 32% increased risk of dementia, heart disease and stroke respectively (6); even it can result in the greater possibility of premature death (5).

Researchers have also studied loneliness of adolescents as they are experiencing developmental transitions biologically and socially (7–10). Specifically, this age group has started to explore friendship and reduce the opportunity of communicating with their parents (11). Such transition from parents to friends may create a big challenge, resulting in an emotional distress (12, 13). In particular, such dis-comfortable or isolation feeling lasted throughout the entire lifespan. Loneliness is also accompanied with other negative emotion like depression and anxiety among adolescents–such co-existing mental health problems are associated with greater probability of committing suicide (14). Thus, the above-mentioned health problems (caused by loneliness) across different age groups could shorten the lifespan as well as bring financial burden to their family.

To this end, potential risk factors for loneliness have attracted great attention from researchers and clinicians. In particular, previous research findings have indicated the potential role of physical or mental illnesses, poor educational level, economically disadvantaged condition, and social isolation (single and living alone) (15). In addition, a study focused on school-aged adolescents, suggesting that parental divorce, family member with physical or mental illness, being bullied, poor student-teacher relationship, excessive use of social media were significantly predictive of being lonely (16). Except for the above-mentioned risk factors, a recent seminal neurological study (14) found that loneliness and hunger (food insecurity refers to the persisting difficulty of accessing to food, leading to disrupted food intake or eating habits) share a home in the brain. Furthermore, previous studies confirmed associations of loneliness with stress-related outcomes (depression, anxiety, and sleep disturbance) but was not linked to food insecurity. In this context, the question is whether loneliness is related to food insecurity. Thus, the aim of this study was to investigate association between food insecurity and loneliness among adolescents across globe.

## Methods

### Study Survey

In this cross-sectional study, the publicly accessible data were retrieved from the GSHS (Details can be found via these Websites: http://www.cdc.gov/gshs; http://www.who.int/chp/gshs) that aimed to identify the modifiable variables contributing to multiple non-infectious illnesses (also known as chronic diseases–including cancer, diabetic, Alzheimer's disease, and mental disorders) in adolescents. Its associated design and sampling procedure are detailed in previous studies (17, 18). In addition, the protocol of this research project was submitted to the National Government Authorities (e.g., the U.S. Department of Education) and their respective ethical review boards, followed by an evaluation process, generating official approval letters for participating researchers and institutions to start data collection. Specifically, participants were invited to complete the consent form before they volunteered to attend this study as well as they were informed that their private information would be kept confidential and anonymous; they were allowed to withdraw at any time if they feel uncomfortable about the question or item being asked in this survey. For data analysis, we selected the latest year/data from each country if they provided more than two datasets. As a result, 68 countries were finally included and they were nationally representative except for several countries where surveys were conducted in selected regions. Given the fact that economical level may be directly linked to food insecurity, income at country level was categorized as low-income, lower- and middle- income, upper-income and middle-income, and high-income, and this variable was controlled in the analysis. Of note, 53 countries were categorized into four groups (based on the World Back Classification) as follow: (1) low-income countries [*n* = 4]; (2) lower- and middle-income countries [*n* = 24]; (3) upper-and middle-income (*n* = 13); (4) high-income countries (*n* = 12). The characteristics of each country are presented in Table S1 (see Supplementary Materials).

### Loneliness (Dependent Variable)

Loneliness as the dependent variable was measured in this study and it involves a single-item question: “During the past 12 months, how often have you felt lonely?”. Five independent options (representing experience of participants: 1 = never, rarely = 2, sometimes = 3, most of the time = 4, and 5 = always) were presented to participants for their selection. For data analysis, these responses were dichotomized into groups: (1) no loneliness [never, rarely and sometimes]; (2) affirmative loneliness [most of the time and always], based on previous studies (19).

### Food Insecurity (Hunger) (Independent Variable)

Food insecurity (hunger) as a predictive variable was measured in this study and it involves a single-item question (2): “During the past 30 days, how often did you go hungry because there was not enough food in your home?” Five independent options (representing experience of participants: 1 = never, rarely = 2, sometimes = 3, most of the time = 4, and 5 = always) were presented to participants for their selection. These responses were in turn grouped into: (1) no food insecurity (never); (2) moderate food insecurity (rarely/sometimes); (3) severe food insecurity (most of the time/always). Specifically, moderate food insecurity indicates compromised food consumption in terms of quality/quantity, while severe food insecurity represents reduced food intake and disrupted eating patterns.

### Controlling Variables

Other variables contributing to loneliness were also extracted in this study in order for more reliable conclusion, including age, sex, physical activity, sedentary behaviors, fruit and vegetable consumption, physical attack, bullying victimization, number of close friends, peer support and parental understanding. These above-mentioned variables were considered as covariates during data analysis in this study.

### Statistical Analysis

For both dependent and predictive variables, overall and country-specific prevalence as descriptive information were computed. To substantiate the association between food insecurity and sleep disturbance (overall and by gender) among adolescents, multivariate logistic regression analyses were conducted while controlling for the above-mentioned variables (gender, age, physical activity, sedentary behavior, bullying victimization, and country). Of note, gender-stratified and country-wise analyses were not adjusted for gender and country, respectively. The value of Higgin's I^2^ that represents the degree of heterogeneity across countries was computed (20) and its associated criteria are presented as follow: (1) <40% = negligible heterogeneity; (2) 40–60% = moderate heterogeneity (21). The pooled estimate was computed based on the random-effects model. A complete case analysis was performed for the cases of missing data. Taylor linearization method was applied to account for the sample weight and clustered study design. Results generating from Logistic regression analyses were presented as odds ratios (ORs) with 95% confidence intervals (CIs). The statistical significance level was set at *p* < 0.05. All statistical analyses were performed using Stata 16.1 (Corp Limited).

## Result

**Figure 1:**
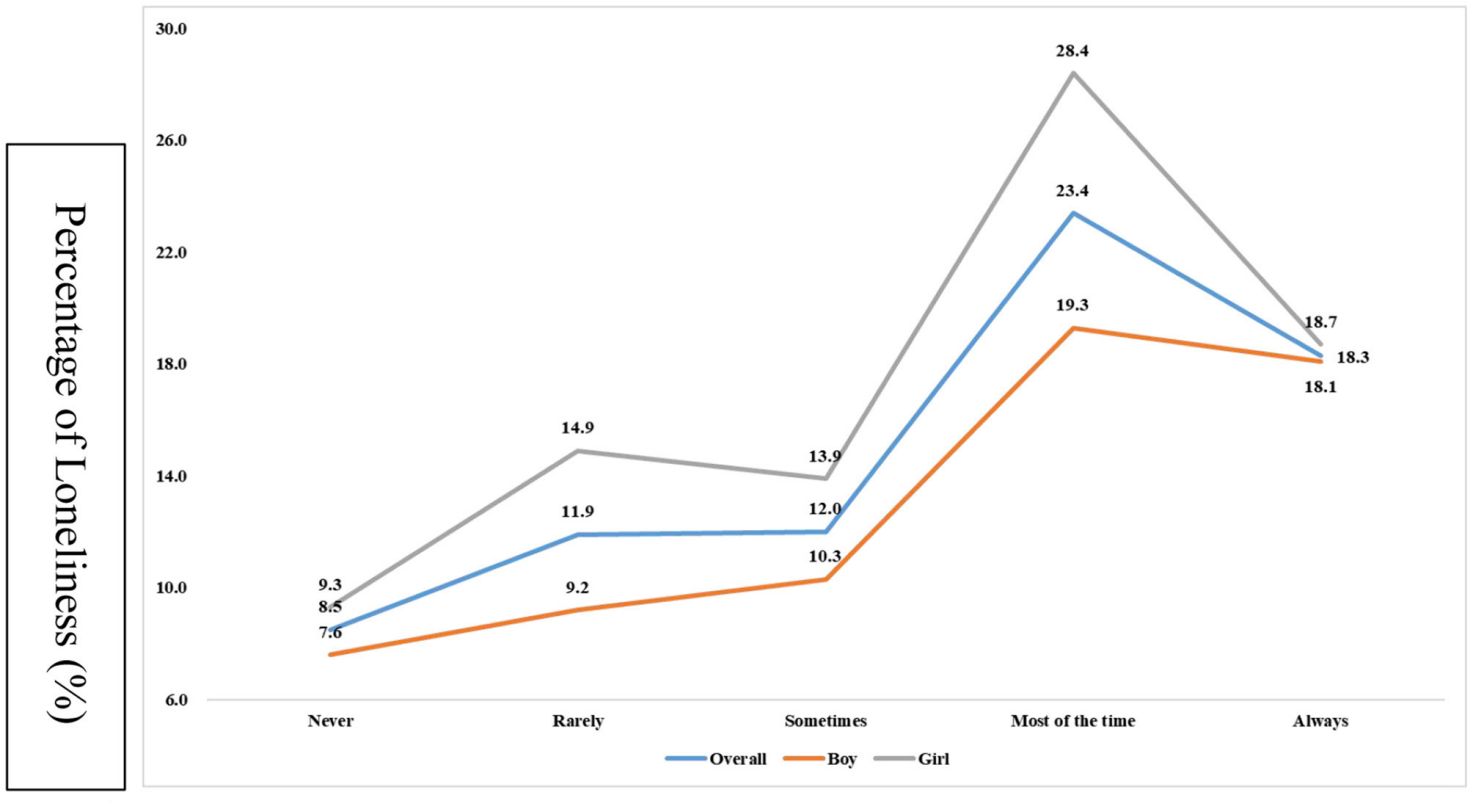
Prevalence of loneliness (%) by levels of food insecurity.

Prevalence in terms of the degrees of food insecurity is reported in Supplementary data. Being most of time and always hungry were 3.3 and 2.3% of the global adolescents (*n* = 164,993). In addition, the prevalence of complaining loneliness has reached 10.8% among these adolescents. The prevalence of loneliness across different severity levels of food insecurity is presented in (Figure 1). Specifically, adolescents with severe food insecurity were linked to greater possibility of reporting loneliness in the overall and gender-stratified samples, as compared with their peers with less severe food insecurity (never/rarely/sometimes).

In the fully adjusted model, Table 1 has shown results on association between food insecurity and loneliness. Adolescents who complained “rarely, sometimes, most of the time, or always hungry” were linked to higher perception of loneliness, as compared to their peers who reported food insecurity. In particular, adolescents who reported severe food insecurity contributed to significantly greater odds for loneliness: (1) being most of the time [OR = 2.54, 95% CI = 2.13–3.02]; (2) always hungry [OR = 1.97, 95% CI = 1.55–2.51]. In addition, a similar result on significant relationship was observed in both boys and girls (Table 1).

**Table 1:** Association between food insecurity and loneliness based on logistic regression model.

	**Overall^a^**			**Boy^b^**			**Girl^b^**		
	**OR**	**95%CI**		**OR**	**95%CI**		**OR**	**95%CI**	
**Never**	**Reference group**								
Rarely	1.21	1.08	1.35	1.06	0.87	1.29	1.36	1.18	1.57
Sometimes	1.40	1.22	1.61	1.32	1.04	1.67	1.49	1.32	1.68
Most of the time	2.54	2.13	3.02	2.32	1.76	3.07	2.80	2.31	3.41
Always	1.97	1.55	2.51	2.20	1.51	3.23	1.81	1.41	2.31

a *Model controlled for age, sex, fruit and vegetable consumption, physically attack, bullying victimization, number of close friends, peer support and parental understanding*.

b *Model controlled for age, sex, fruit and vegetable consumption, physically attack, bullying victimization, number of close friends, peer support and parental understanding except for sex*.

**Figure 2:**
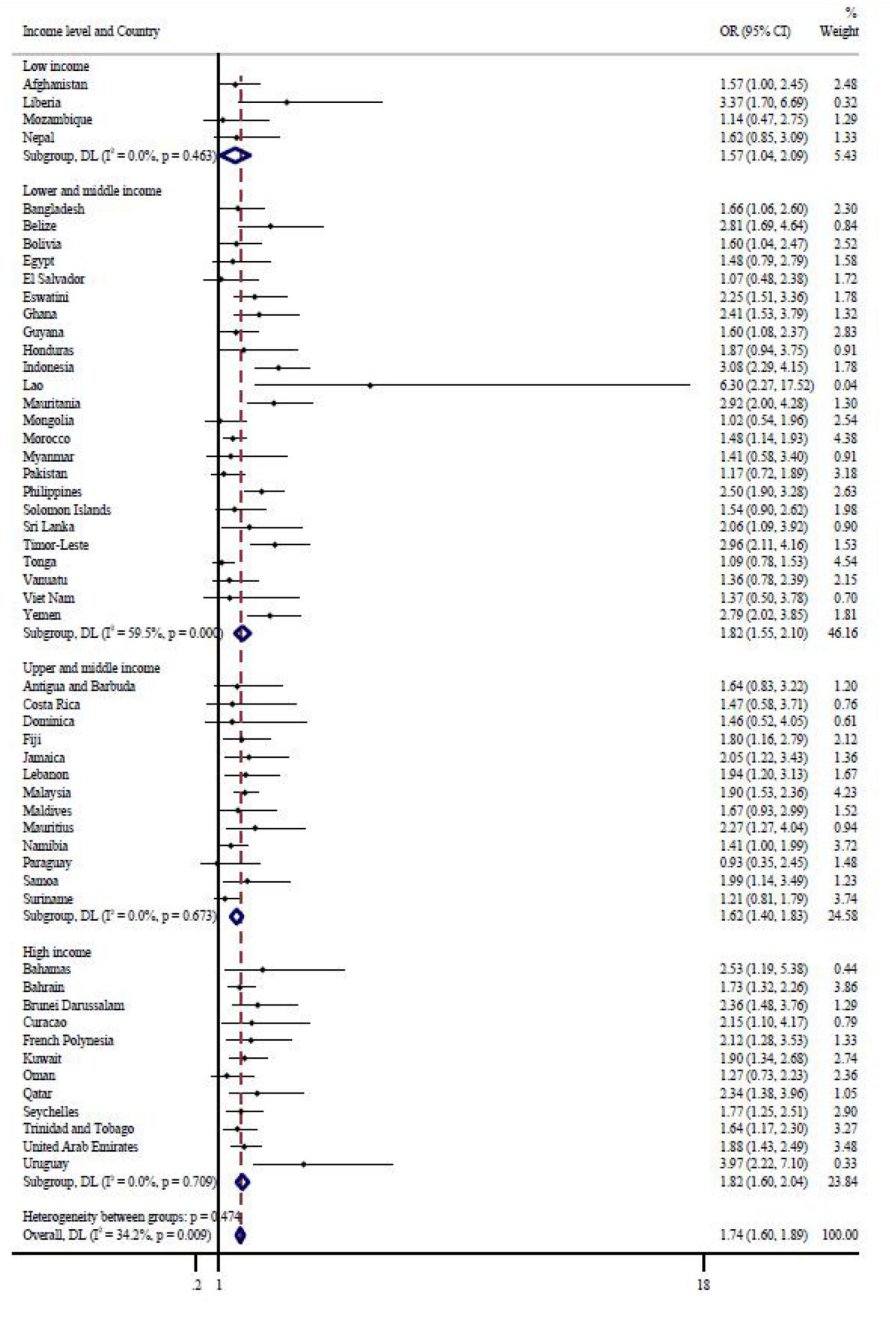
Country-wise association between severe food insecurity (most of the time/always hungry) and loneliness estimated by multivariable logistic regression. OR, Odds ratio; CI, Confidence interval. Reference category is no food insecurity (never, rarely, sometimes). Models are adjusted for age, sex, fruit and vegetable consumption, physically attack, bullying victimization, number of close friends, peer support and parental understanding. Overall estimates were obtained by meta-analysis with random effects.

Figure 2 shows results in terms of country-wise multivariate logistic regression analysis. adolescents who were always hungry or in most the time (representing severe food insecurity) were significantly linked to an increased risk of perceiving loneliness in 39 of the 53 included countries, as compared to less severe food insecurity (never/rarely/sometimes). The pooled OR is 1.74 (1.60–1.89), with the country-based I-square of 34.2% (negligible heterogeneity). Higher I-squared value (I^2^ = 59.5%, representing moderate heterogeneity) was observed in lower- and middle-income countries while investigating the association between food insecurity and loneliness.

## Discussion

### Main Findings

With respect to the role of food insecurity in emotional regulation, to our knowledge, the current study is the first time to investigate the association between food insecurity and loneliness among adolescents, especially in a global sample. Being most of time and always hungry were 3.3 and 2.3% of the global adolescents, while the prevalence of complaining loneliness was 10.8%. In addition, results indicated that 41.7% of adolescents with loneliness complained with severe food insecurity (reflected by most of time and always)–the number is relatively smaller in other three groups (never, rarely, and sometime). In other words, the prevalence of loneliness has become greater as the degree of food insecurity elevated. With the adjustment of covariates, being hungry in most of time has the greatest likelihood of complaining loneliness, followed by being always hungry, which is independent of sexes (boys and girls). Furthermore, serious food insecurity was observed to associate, of great significance, with a higher likelihood of complaining loneliness in 39 of 53 economies within the global studying sample, with overall OR being 1.74 (1.60–1.89, I^2^ = 34.2%, negligible heterogeneity). Taking closer into income-based sub-group analysis, significant association between food insecurity and loneliness was only observed in lower- and middle-income countries, with great degree of heterogeneity.

### Interpretation of Findings

Approximately 46% of adolescents reported moderate food insecurity. Such number is relatively higher as compared to a previous study based on the Global School-based Student Health Survey, but the severe food insecurity is relatively smaller in the current study (22). Accumulating evidence indicate that the negative effects of food insecurity on both psychological and physical measures, which may be attributed to malnutrition (lack of nutrient intake). Such impairment has become more serious as adolescents experience developmental transition in a relatively rapid speed, which specifically resulted in short height (greater BMI–obesity), neuro-cognitive impairment, sleep disturbance (23), leading to poor academic performance at school and worse social relationship. Such public health issue requires greater attention.

Investigation on association between food insecurity and loneliness, based on this global sample, can be confirmed. Previous studies mainly focused on association of food insecurity with other mental health measures including stress, depression, anxiety, suicidal behaviors (24–26), and sleep condition. To this end, our results in the present study adds to existing literature. From the biological perspective, a previous study, especially those relating to brain science, have come up with the conclusion that the part of the brain lighting up in the condition of hungry shares the same part in active as that of the situation of feeling lonely, thus may, to a certain extent, imply the correlation between food insecurity and loneliness, somehow could as well be considered as supporting materials indicating the association between food insecurity and loneliness. If understanding the findings from the standpoint of contextual factors, when talking about food security and one direct consequence, hunger, lack of money may always be the first thought. Indeed, shortage of living resources caused by poverty may not directly lead to the condition of isolation and loneliness, yet bare livelihood would undoubtedly result in dilemma, especially when facing necessity in respect of various kinds of expenditure, such as housing loan, rent, children's education fee and health care fee. It is not difficult to foresee that such difficulties may, of great possibility, aggravate the feeling of helplessness, which may probably lead to and increase the degree of loneliness.

Overall speaking, the heterogeneity of the correlation between food insecurity and loneliness was negligible, indicating that the correlation between food insecurity and loneliness was not something rare in individual cases, it, instead, was common among adolescents within the sample of selected economies (different levels of income). Yet, large difference concerning the association between food insecurity and loneliness was found especially in the category of lower- and middle-income economies, which could be understood from such perspective that even falling into the same scope, social determinants influencing the degree of food insecurity, including but not limited to economy, culture, environment and related policies, would vary, probably to a large extent, the relationship among these factors and food insecurity may still need to be further clarified so as to accordingly look deeper into the association between food insecurity and loneliness.

### Implications for Practice

In addition to the results, corresponding analysis indeed implied the prevalence of food insecurity (27) and loneliness (28) among adolescents, thus it would be helpful in providing one perspective to understand the correlation between such two issues and to accordingly put forward targeted plans and strategies plus carrying out effective measures, including but not limited to the increase of continuous capital investment and the establishment of proper coordination mechanisms concerning food supplies to students, especially to those from families that are shortage of most basic food security. The findings of the possible role of food insecurity in forming loneliness as well-indicates the necessity of more detailed researches on hunger-caused loneliness and mental health protection and intervention. Specifically, together with persistent researches concerning brain science and respective working mechanisms, especially those relating to neuroscience, well-directed analysis and assessment need to be carried out, with the inclusion of more factors such as eating habits.

### Limitations and Strength

Indeed, compared with existing observations (26, 29), this study, instead of only focusing on one or few economies with a relatively small sample, paid attention to a larger sample of adolescents from 53 economies of various income levels so as to look into the relationship between food insecurity and loneliness, providing a relatively wider perspective of understanding in respect of influence factors of loneliness. Despite such advantage, some limitations of this study need to be admitted. Firstly, the data from GSHS was collected based on self-reporting, which means that recall bias and desirability bias would be possible to some extent. Secondly, as the data adopted were cross-sectional, longitudinal study, in addition to current research perspective, might still be conducted in case the association between food insecurity and loneliness was uneasy to clearly establish. Thirdly, as previously noted, the level of food loneliness was decided by the frequency of hunger, yet such aspect may not be sufficient to come up with a comprehensive picture of food loneliness. Fourth, loneliness in this study mainly refers to the kind probably caused by hunger, thus the finding applicable to other kinds of loneliness should be made with caution.

## Conclusion

Food insecurity has found to be prevalent among adolescents and to be positive correlation with the level of loneliness within the economies of this study. As food insecurity, especially with the continuous epidemic globally, tends to be more severe, it is not difficult to foresee that loneliness would become one of the global mental problem, which urgently require effective and promptly resolutions, including but not limited to targeted policies and measures with official and unofficial efforts plus international cooperation and coordination among developed and developing countries.

## Data Availability Statement

The original contributions presented in the study are included in the article/Supplementary Material, further inquiries can be directed to the corresponding author.

## Ethics Statement

The studies involving human participants were reviewed and approved by Centers for Disease Control and Prevention (CDC) and World Health Organization (WHO). Written informed consent to participate in this study was provided by the participants' legal guardian/next of kin.

## Author Contributions

HW: manuscript writing-original draft preparation, methodology, and analysis. TG: supervision and validation. ZG and LZ: editing. All authors: review and editing. All authors contributed to the article and approved the submitted version.

## Funding

This work was supported by grant of National Natural Science Foundation of China (No. 31871115).

## Conflict of Interest

The authors declare that the research was conducted in the absence of any commercial or financial relationships that could be construed as a potential conflict of interest.

## Publisher's Note

All claims expressed in this article are solely those of the authors and do not necessarily represent those of their affiliated organizations, or those of the publisher, the editors and the reviewers. Any product that may be evaluated in this article, or claim that may be made by its manufacturer, is not guaranteed or endorsed by the publisher.
