# Supplementary material for: Do Global Adolescents With Food Insecurity Feel Lonely?
Source: Front Public Health. 2022 Feb 10;10:820444. doi: 10.3389/fpubh.2022.820444 (PMC8868937; doi:10.3389/fpubh.2022.820444)
Supplement: Supplementary file 1 [file Table_1.docx]

**Table S1. Sample characteristics and prevalence of food insecurity and loneliness.**

| **Country** |  | **Sample size** |  | **Food insecurity (%)** | | | | |  | **Loneliness (%)** |
| --- | --- | --- | --- | --- | --- | --- | --- | --- | --- | --- |
|  |  |  |  | **Never** | **Rarely** | **Sometimes** | **Most of the time** | **Always** |  |  |
| **Afghanistan** |  | 1543 |  | 49.2 | 12.2 | 17.7 | 15.0 | 5.9 |  | 31.4 |
| **Antigua and Barbuda** |  | 1031 |  | 56.1 | 14.3 | 23.0 | 4.2 | 2.4 |  | 12.4 |
| **Bahamas** |  | 1104 |  | 53.1 | 18.8 | 21.9 | 4.9 | 1.3 |  | 10.6 |
| **Bahrain** |  | 6482 |  | 44.2 | 22.9 | 22.1 | 8.6 | 2.2 |  | 15.4 |
| **Bangladesh** |  | 2635 |  | 37.9 | 8.2 | 41.0 | 6.2 | 6.6 |  | 10.0 |
| **Belize** |  | 1405 |  | 47.4 | 23.3 | 15.1 | 5.3 | 8.8 |  | 12.2 |
| **Bolivia** |  | 3109 |  | 38.9 | 34.9 | 17.9 | 3.7 | 4.5 |  | 10.5 |
| **Brunei Darussalam** |  | 2312 |  | 38.2 | 30.0 | 25.5 | 5.0 | 1.4 |  | 12.1 |
| **Costa Rica** |  | 2451 |  | 80.1 | 12.7 | 6.0 | 0.7 | 0.5 |  | 6.4 |
| **Curacao** |  | 1862 |  | 75.9 | 12.0 | 8.7 | 2.3 | 1.1 |  | 15.7 |
| **Dominica** |  | 1098 |  | 64.9 | 20.5 | 12.9 | 0.8 | 0.8 |  | 14.0 |
| **Egypt** |  | 2019 |  | 54.3 | 25.4 | 16.4 | 3.2 | 0.7 |  | 14.5 |
| **El Salvador** |  | 1652 |  | 66.2 | 19.7 | 11.0 | 1.4 | 1.7 |  | 8.7 |
| **Eswatini** |  | 2313 |  | 48.6 | 6.9 | 37.1 | 5.4 | 2.0 |  | 9.1 |
| **Fiji** |  | 2590 |  | 42.0 | 9.5 | 38.2 | 8.2 | 2.1 |  | 13.5 |
| **French Polynesia** |  | 2536 |  | 36.2 | 32.9 | 21.0 | 7.7 | 2.2 |  | 10.2 |
| **Ghana** |  | 1221 |  | 36.2 | 5.9 | 44.0 | 7.9 | 6.0 |  | 12.5 |
| **Guyana** |  | 2058 |  | 55.1 | 12.5 | 25.3 | 4.0 | 3.1 |  | 17.6 |
| **Honduras** |  | 1529 |  | 64.0 | 22.2 | 9.8 | 1.8 | 2.3 |  | 10.5 |
| **Indonesia** |  | 9719 |  | 45.2 | 12.4 | 38.6 | 2.6 | 1.2 |  | 6.1 |
| **Jamaica** |  | 1381 |  | 52.1 | 21.4 | 20.7 | 4.2 | 1.6 |  | 18.3 |
| **Kuwait** |  | 2639 |  | 52.3 | 22.9 | 18.2 | 5.0 | 1.6 |  | 17.8 |
| **Lao** |  | 3281 |  | 51.1 | 15.7 | 32.0 | 0.9 | 0.3 |  | 3.7 |
| **Lebanon** |  | 4396 |  | 71.0 | 18.5 | 8.1 | 1.7 | 0.7 |  | 10.8 |
| **Liberia** |  | 865 |  | 39.9 | 1.9 | 46.7 | 8.7 | 2.9 |  | 10.1 |
| **Malaysia** |  | 23224 |  | 40.8 | 28.3 | 26.4 | 2.4 | 2.1 |  | 7.6 |
| **Maldives** |  | 2546 |  | 64.9 | 13.8 | 16.1 | 3.8 | 1.4 |  | 15.8 |
| **Mauritania** |  | 1629 |  | 40.6 | 31.7 | 18.0 | 5.2 | 4.5 |  | 14.3 |
| **Mauritius** |  | 2600 |  | 57.9 | 19.1 | 16.3 | 4.4 | 2.3 |  | 10.2 |
| **Mongolia** |  | 4798 |  | 63.5 | 22.9 | 12.1 | 0.8 | 0.7 |  | 12.0 |
| **Morocco** |  | 4827 |  | 67.8 | 11.7 | 12.0 | 6.0 | 2.5 |  | 18.2 |
| **Mozambique** |  | 1067 |  | 54.5 | 9.1 | 26.0 | 4.3 | 6.1 |  | 9.3 |
| **Myanmar** |  | 2488 |  | 69.4 | 6.3 | 22.1 | 1.1 | 1.0 |  | 8.2 |
| **Namibia** |  | 2755 |  | 48.3 | 5.5 | 36.9 | 6.5 | 2.7 |  | 14.5 |
| **Nepal** |  | 5433 |  | 68.2 | 4.9 | 23.1 | 2.1 | 1.6 |  | 5.8 |
| **Oman** |  | 2713 |  | 64.3 | 17.4 | 14.2 | 2.7 | 1.3 |  | 14.7 |
| **Pakistan** |  | 4365 |  | 75.4 | 6.3 | 13.1 | 2.1 | 3.1 |  | 11.5 |
| **Paraguay** |  | 2575 |  | 71.9 | 17.5 | 8.6 | 0.9 | 1.1 |  | 10.5 |
| **Philippines** |  | 7674 |  | 30.2 | 32.4 | 30.3 | 4.4 | 2.7 |  | 15.8 |
| **Qatar** |  | 1267 |  | 71.8 | 14.8 | 7.8 | 3.6 | 2.0 |  | 16.0 |
| **Samoa** |  | 1432 |  | 25.6 | 36.3 | 25.7 | 5.0 | 7.4 |  | 8.7 |
| **Seychelles** |  | 2053 |  | 56.8 | 15.9 | 15.6 | 7.2 | 4.4 |  | 11.9 |
| **Solomon Islands** |  | 1085 |  | 15.6 | 8.6 | 65.6 | 7.5 | 2.7 |  | 13.9 |
| **Sri Lanka** |  | 3042 |  | 72.1 | 13.0 | 12.0 | 1.0 | 1.9 |  | 8.5 |
| **Suriname** |  | 1717 |  | 57.7 | 11.3 | 20.2 | 5.4 | 5.4 |  | 16.5 |
| **Timor-Leste** |  | 2356 |  | 50.6 | 13.7 | 25.4 | 4.4 | 6.0 |  | 12.6 |
| **Tonga** |  | 2816 |  | 34.6 | 25.2 | 29.1 | 4.2 | 6.9 |  | 13.5 |
| **Trinidad and Tobago** |  | 3078 |  | 49.5 | 22.5 | 20.4 | 5.3 | 2.4 |  | 14.4 |
| **United Arab Emirates** |  | 4750 |  | 54.2 | 20.0 | 17.9 | 6.0 | 1.9 |  | 13.0 |
| **Uruguay** |  | 3142 |  | 77.0 | 16.1 | 5.5 | 0.9 | 0.5 |  | 7.1 |
| **Vanuatu** |  | 1688 |  | 38.8 | 4.4 | 49.0 | 5.0 | 2.8 |  | 9.6 |
| **Viet Nam** |  | 2801 |  | 48.1 | 30.6 | 20.3 | 0.5 | 0.5 |  | 11.2 |
| **Yemen** |  | 1841 |  | 43.7 | 24.6 | 21.6 | 7.8 | 2.3 |  | 15.5 |
| **Total** |  | 164993 |  | 48.8 | 18.6 | 27.0 | 3.3 | 2.3 |  | 10.8 |
